# Association between ischemic stroke and hyperintense plaques detected by high-resolution vessel wall MRI in Japanese patients with intracranial atherosclerotic disease

**DOI:** 10.3389/fstro.2025.1610666

**Published:** 2025-07-22

**Authors:** Naoki Takayama, Takakuni Maki, Yasutaka Fushimi, Masakazu Okawa, Yohei Mineharu, Kiyofumi Yamada, Tao Yang, Yu Yamamoto, Keita Suzuki, Ken Yasuda, John Grinstead, Sinyeob Ahn, Riki Matsumoto, Yoshiki Arakawa, Kazumichi Yoshida

**Affiliations:** ^1^Department of Neurosurgery, Kyoto University Graduate School of Medicine, Kyoto, Japan; ^2^Department of Neurology, Kyoto University Graduate School of Medicine, Kyoto, Japan; ^3^Department of Diagnostic Imaging and Nuclear Medicine, Kyoto University Graduate School of Medicine, Kyoto, Japan; ^4^Department of Neurosurgery, Hyogo College of Medicine Hospital, Kyoto, Japan; ^5^Siemens Healthineers USA, Malvern, PA, United States; ^6^Department of Neurosurgery, Shiga University of Medical Science, Kyoto, Japan

**Keywords:** hyperintense plaque, intracranial atherosclerotic disease, stroke, high-resolution vessel wall imaging, DANTE T1-SPACE

## Abstract

**Background:**

Conventional MRI sequences are insufficient for the detailed depiction of intracranial atherosclerotic disease (ICAD) plaques. The aim of this study was to investigate the association between ischemic events and intracranial atherosclerotic plaque characteristics using a high-resolution T1-weighted black-blood MRI technique (DANTE T1-SPACE) in the anterior circulation in the Japanese population.

**Methods:**

Patients with a total of 108 lesions causing ≥40% stenosis on the C1–5 segments of the intracranial internal carotid artery (ICA) or M1 segment of the middle cerebral artery (MCA) were included. Hyperintense plaques (HIPs) were defined as plaques with a spot of signal intensity (SI) higher than 1.5-fold SI of the ipsilateral temporal muscle on DANTE T1-SPACE. The vessel wall lesions were divided into symptomatic and asymptomatic groups. The lesions in the symptomatic group were classified as artery-to-artery embolism, hemodynamic infarction, cardiac embolism, undetermined and transient ischemic attack (TIA).

**Results:**

Among the 108 plaques, 19 were symptomatic and 89 were asymptomatic. The percentage of HIPs in the symptomatic group was significantly higher than in the asymptomatic group (57.9% vs. 24.7%, *p* = 0.01). In the symptomatic group, the proportion of HIPs in the A-to-A embolism subgroup was higher than in the other subgroups.

**Conclusions:**

DANTE T1-SPACE may aid in the identification of intracranial plaques with imaging characteristics suggestive of increased stroke risk, particularly hyperintensity potentially reflecting intraplaque hemorrhage.

## Introduction

Several studies on cervical carotid artery stenosis have demonstrated the association between ischemic events and plaque characteristics, particularly intraplaque hemorrhage (IPH), detected as a hyperintensity on T1-weighted MRI (Takaya et al., 2006; Yamada et al., 2007; Altaf et al., 2007, 2008; Kurosaki et al., 2011). Although intramural plaques of intracranial atherosclerotic disease (ICAD) are also considered one of the major causes of ischemic strokes (Wong, 2006), conventional MRI sequences without dedicated vessel wall protocols are often insufficient to visualize these plaques due to the small caliber and complex geometry of intracranial arteries. While high-resolution T1- and T2-weighted sequences can partially visualize and characterize intracranial plaques, specialized vessel wall imaging (VWI) techniques provide improved spatial resolution and contrast for more accurate plaque assessment (Mossa-Basha et al., 2016). Recent technical improvements in VWI with high-resolution MRI (HR-MRI) allowed the depiction of intracranial vessel walls and consequently, reports on the characterization of ICAD plaques have been increasing (Mandell et al., 2017).

T1-weighted three-dimensional (3D) variable refocusing flip angle (VRFA) turbo spin-echo sequence known as sampling perfection with application-optimized contrast using different flip angle evolutions (SPACE) is a commonly used intracranial VWI (IVWI) technique. This sequence enables isotropic, submillimeter-resolution imaging of intracranial vessel walls with broad anatomical coverage and the ability to reconstruct images in multiple planes. It also provides an inherent black-blood (BB) effect that helps suppress intraluminal blood signal and enhances plaque visualization (Mossa-Basha et al., 2016). While T1-weighted SPACE sequences are generally effective for intracranial vessel wall imaging, residual flow-related signal may persist in regions of slow or complex flow, such as the anterior and posterior genu of the carotid siphon. This residual signal can sometimes be misinterpreted as plaque (Xie et al., 2016). The addition of delay alternating with nutation for tailored excitation (DANTE) preparation pulses helps overcome this limitation by further suppressing slow and turbulent flow signals, thereby enhancing the black-blood effect and improving plaque delineation (Wang et al., 2016). DANTE pulses attenuate flow-related signal components, improving suppression of residual blood and CSF signal, thereby enhancing vessel wall contrast and delineation. Incorporating DANTE pulses into T1-weighted VRFA allows a more detailed evaluation of vessel walls than T1-weighted VRFA alone (Mossa-Basha et al., 2016; Xie et al., 2016; Fushimi et al., 2022). Hyperintense plaques (HIPs) are defined as atherosclerotic plaques containing focal regions with high signal intensity on T1-weighted vessel wall images, typically observed using black-blood MRI techniques such as DANTE T1-SPACE. These hyperintense areas are considered to reflect intraplaque hemorrhage (IPH), a hallmark of plaque vulnerability. In extracranial carotid arteries, histopathological correlation has demonstrated that T1 hyperintensity corresponds to IPH and is associated with symptomatic lesions and soft plaque components (Yoshida et al., 2008). Although direct histological validation is limited in the intracranial circulation, HIPs are similarly regarded as indicators of unstable plaques with increased risk for rupture or thrombosis. Thus, detection of HIPs may serve as a non-invasive imaging biomarker of intracranial plaque instability and stroke risk.

Intracranial atherosclerotic disease (ICAD) is a major cause of ischemic stroke, accounting for up to 50% of cases in Asia (Wong, 2006). However, most vessel wall MRI studies have focused on Chinese populations, with limited data available for other ethnic groups, including Japanese patients. This study aimed to evaluate the association between ischemic stroke and the presence of HIPs in a Japanese cohort, using high-resolution black-blood imaging with DANTE T1-SPACE. By applying this advanced imaging technique, we investigated whether HIPs are more frequently observed in symptomatic plaques. Given its superior flow suppression—particularly in tortuous segments like the carotid siphon—DANTE preparation enhances detection of subtle vessel wall abnormalities that may be overlooked using conventional methods. Although this study focused on the anterior circulation due to superior image quality and reproducibility in those regions, the findings may offer insights into stroke risk stratification and guide future research across diverse populations.

## Methods

### Study population

From August 2016 to August 2018, a total of 1,244 MRI examinations that included an IVW imaging using Siemens prototype sequence, DANTE T1-SPACE were performed in our hospital. We retrospectively selected the subjects with ≥ 40% stenosis on the C1–5 segments of the ICA or the M1 segment of the MCA. The C1–C5 segments of the ICA were defined according to the Bouthillier classification system (Bouthillier et al., 1996), which anatomically categorizes the ICA from its petrous to communicating segments. When multiple MRI examinations were performed during the same period for the same subject, only the first scan was evaluated. In cases with multiple ipsilateral stenoses, the most severe stenosis was selected. If stenoses were found bilaterally, both lesions were assessed. The exclusion criteria were as follows: (1) previous history of endovascular therapy, such as mechanical thrombectomy or percutaneous transluminal angioplasty; (2) stenosis due to causes other than atherosclerosis, such as angiitis, dissection, and reversible cerebral vasoconstriction syndrome; (3) coexistence with ≥50% stenosis of the extracranial carotid artery; and (4) previous history of carotid endarterectomy or carotid artery stenting on the ipsilateral side. Age, sex, stenosis percentage, and lesion location were recorded for all cases. For symptomatic cases, MRI including DANTE T1-SPACE was performed within 14 days from stroke onset or TIA symptoms, ensuring relevance to acute-phase plaque evaluation. This retrospective study was approved by the local institutional review board (approval number: R1359-2), and written informed consent was waived.

### MRI protocol

MRI scanning was performed using 3T MRI scanners (MAGNETOM Skyra or Prisma; Siemens Healthineers, Erlangen, Germany) with a 32-channel head coil or 64-channel head/neck coil. For all patients, MRI included diffusion-weighted imaging (DWI) and three-dimensional time-of-flight magnetic resonance angiography (3D TOF MRA). In symptomatic cases, cerebral blood flow (CBF) was also evaluated using single-photon emission computed tomography (SPECT). 3D DANTE T1-SPACE was acquired with the following parameters: TR/TE, 1,000/11 ms; FOV, 180 × 180 mm; matrix, 320 × 320; slice thickness, 0.56 mm (0.56 mm isotropic voxel); receiver bandwidth, 380 Hz/pixel; acceleration factor, 2 × 2. The details of the DANTE pulses were as follows: pulse trains 148; RF-pulse flip angle, 10°; pulse spacing, 1.1 ms, with a spoiler gradient applied along the three axes. The acquisition time was 5 min 44 s.

### Image analysis

The stenosis percentage was calculated using the MRA maximum intensity projection based on the Warfarin-Aspirin Symptomatic Intracranial Disease (WASID) method, with the following formula: stenosis percentage = (1 – D_stenosis_/D_normal_) × 100 where D_stenosis_ is the lowest artery diameter, and D_normal_ is the diameter of the proximal normal artery. We defined significant stenosis as ≥40% diameter reduction by the WASID method, which roughly corresponds to ≥60% area stenosis (Xu et al., 2012; Yang et al., 2014; Wu et al., 2018). This criterion was selected to capture a clinically relevant range of intracranial atherosclerosis while ensuring consistency with prior MRA-based studies. Signal measurements were performed using standardized, manually placed circular regions of interest (ROIs) with diameters of 3–5 mm. ROIs were placed at the brightest area of the plaque and within the ipsilateral temporal muscle, avoiding partial volume effects and adjacent structures. The SI of the brightest spot of a plaque (SI_plaque_) and ipsilateral temporal muscle (SI_muscle_) were measured using DANTE T1-SPACE. When SI_plaque_/SI_muscle_ was >1.5, the plaque was defined as a HIP. The threshold of 1.5 × signal intensity was adopted based on prior studies (Xu et al., 2012; Yang et al., 2014; Wu et al., 2018), which defined hyperintense plaques using similar criteria to reflect possible intraplaque hemorrhage. This standardization enhances reproducibility across studies using different MRI protocols. Interrater reliability between two examiners (NT and TM) was assessed using quantitative measurements obtained for 20 plaques. Five parameters were measured by both raters: plaque intensity, mean signal intensity, adjacent muscle signal intensity, and two derived signal intensity ratios (Ratio J and Ratio K). For each parameter, the agreement between raters was evaluated using: intraclass correlation coefficient (ICC) based on a two-way random-effects model for absolute agreement [ICC(2,1)]. All analyses were performed using Python (version 3.12.7) with the pingouin, scipy, and matplotlib packages. The ICCs for plaque intensity, mean signal intensity, adjacent muscle signal intensity, Ratio J and Ratio K were 0.980, 0.980, 0.984, 0.926, and 0.875, respectively, demonstrating excellent inter-observer reliability for most parameters and good reliability for Ratio K.

### Lesion classification

**Figure 1:**
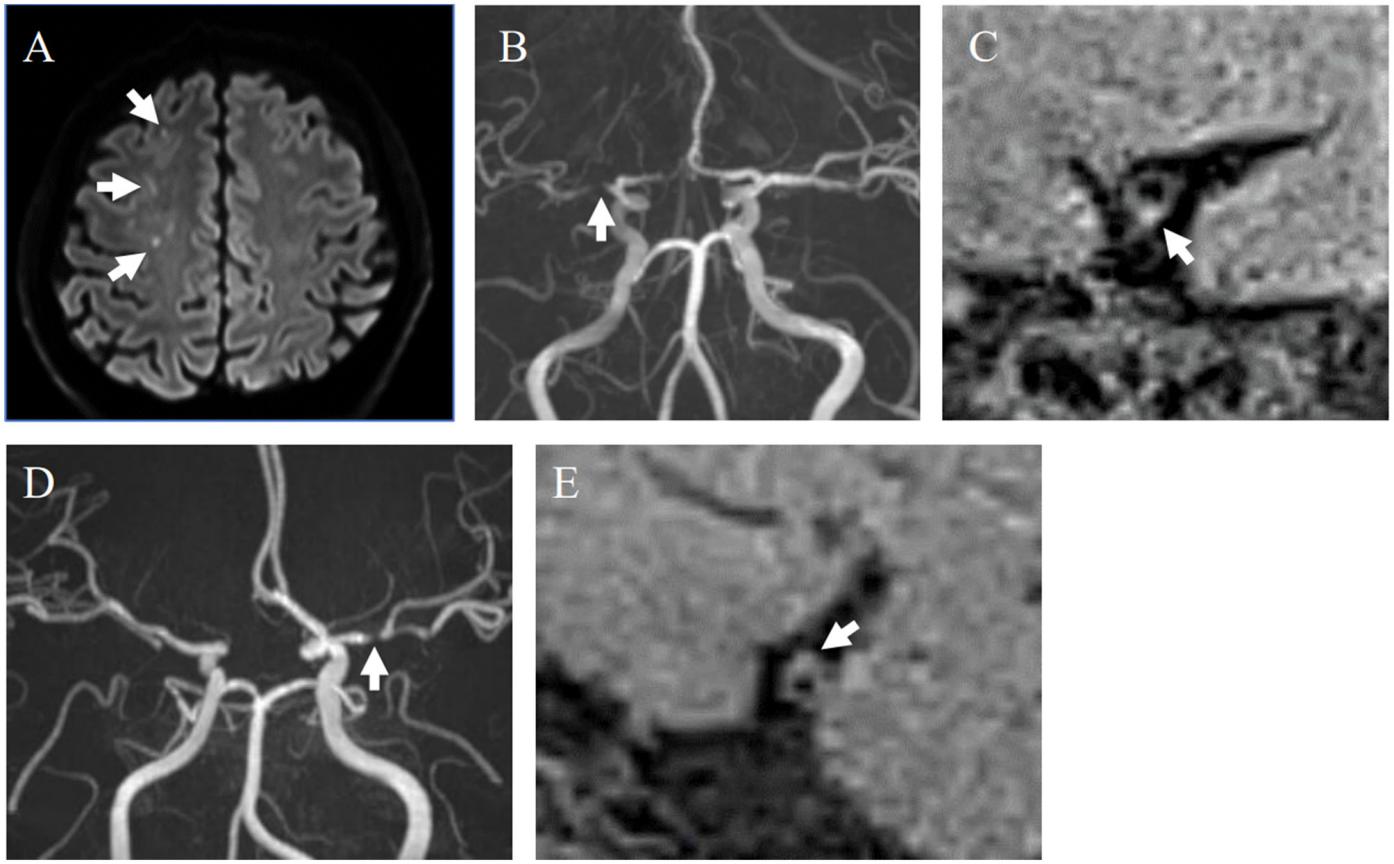
Representative cases with and without HIPs. **(A–C)** A 40-year-old male with symptomatic stenosis in the right MCA. **(A)** Multiple cerebral infarcts were detected in the right cerebral white matter on DWI (white arrows). **(B)** MRA showed severe stenosis of the M1 segment of the right MCA (white arrow). **(C)** An HIP was detected on the sagittal section of the MCA on DANTE T1-SPACE (white arrow). SIplaque/SImuscle ratio was 1.61. This case was diagnosed as A-to-A embolism. **(D, E)** 59-year-old female with asymptomatic stenosis in the left MCA. **(D)** MRA showed severe stenosis of the M1 segment of the left MCA (white arrows). **(E)** The plaque without hyperintensity was detected on the sagittal section of the MCA on DANTE T1-SPACE. SIplaque/SImuscle ratio was 1.33.

All plaques were divided into symptomatic and asymptomatic groups. A plaque was considered symptomatic when it satisfied one of the following criteria: (1) presence of a hyperintense area on DWI within the territory supplied by the stenotic artery; (2) transient ischemic attack (TIA) with symptoms associated with the cerebral region supplied by the stenotic artery within the 4 weeks preceding the MRI examination, with no acute ischemic lesions detected on DWI. Furthermore, the symptomatic cases were subdivided into five subgroups: (a) artery-to-artery (A-to-A) embolism, defined as more than two hyperintense lesions on DWI detected in the territory supplied by the stenotic artery. Moreover, SPECT showed that the resting CBF in this territory was >80% of the normal value, atrial fibrillation (AF) was not detected by an electrocardiogram (ECG), and transthoracic echocardiography showed no intracardiac thrombi. (b) Hemodynamic infarction, defined as more than two hyperintense lesions on DWI detected in the territory supplied by the stenotic artery, with stenosis ≥70%, SPECT showing resting CBF in the territory <80% of the normal value, and cardiac embolism was excluded as described above. (c) Perforator infarction: a single hyperintense lesion on DWI detected in the perforating artery territory (internal capsule, basal ganglia, and corona radiata) supplied by the stenotic artery, and stenosis located proximally to the bifurcation of the perforator. (d) Undetermined lesions: hyperintense lesions detected on DWI when the first three cases were excluded. This category included suspected cases of cardiac embolism, in which AF was detected on ECG, according to the TOAST classification (Adams et al., 1993). (e) TIA cases without DWI lesions were in the fifth subgroup same as (2) above. The proportion of HIPs was calculated for the overall symptomatic and asymptomatic groups, as well as for each of the five subtypes within the symptomatic group. Two representative cases with and without a HIP are shown in Figure 1.

### Statistical analysis

Quantitative data are expressed as mean ± standard deviation and qualitative data are expressed as counts and percentages. Continuous variables were compared using the Mann–Whitney *U*-test, and categorical variables were analyzed using Fisher's exact test. Statistical significance was set at *P* < 0.05. JMP^®^ 14 (SAS Institute Inc., Cary, NC, The United States) was used for the statistical analysis.

## Results

**Figure 2:**
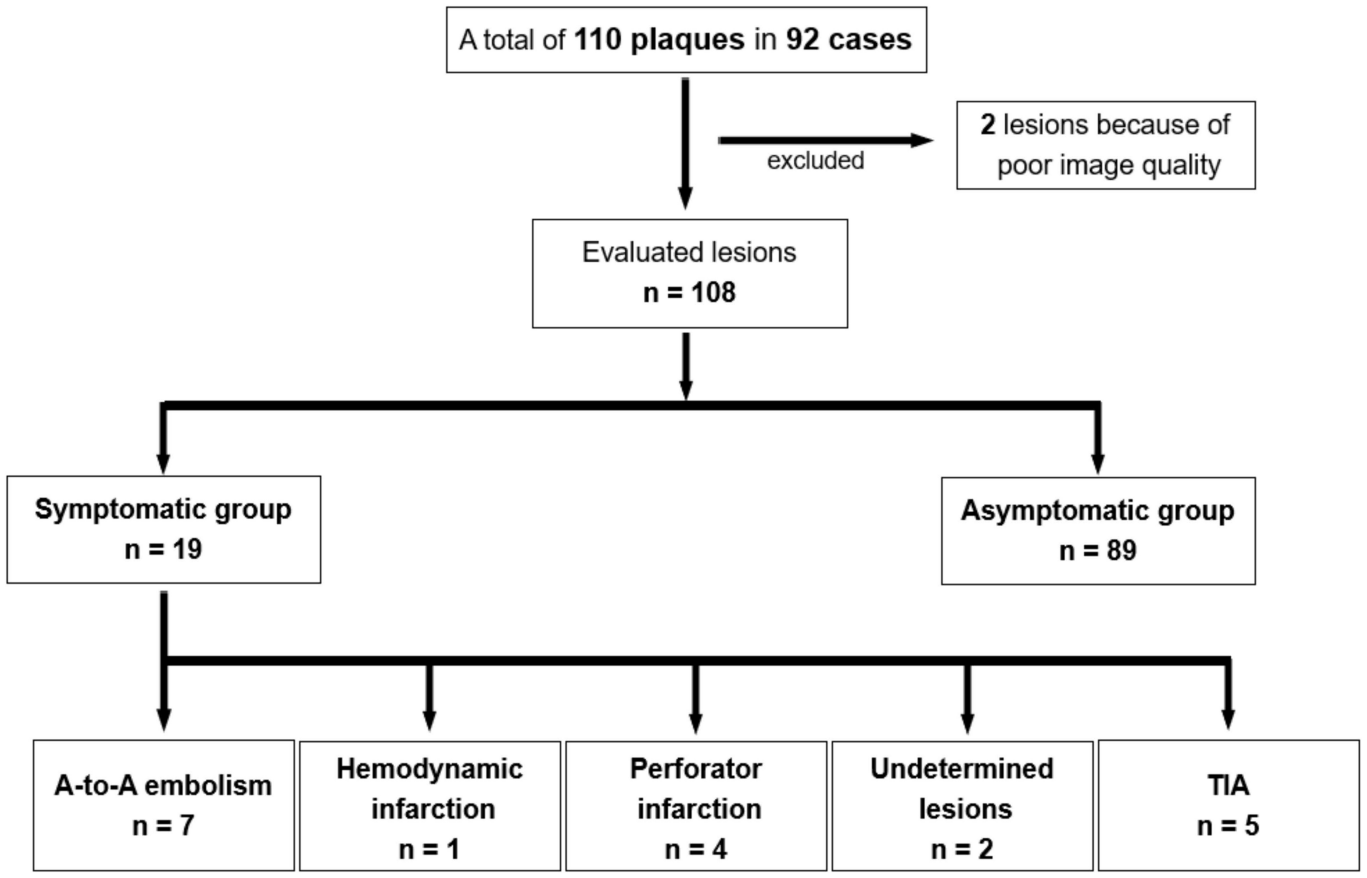
The flowchart of this study.

A total of 110 plaques in 92 patients were included in the analysis. Among the 110 plaques, two lesions were excluded due to due to motion artifact in DANTE T1-SPACE images; remaining 108 plaques were analyzed. Among these plaques, 19 were symptomatic and 89 were asymptomatic. The symptomatic lesions were subdivided as follows: A-to-A embolism (*n* = 7); hemodynamic infarction (*n* = 1); perforator infarction (*n* = 4); undetermined (*n* = 2); and TIA (*n* = 5, Figure 2).

The clinical characteristics and locations of the lesions are shown in Table 1. No statistically significant differences were observed regarding sex, age, and stenosis percentage between the symptomatic and asymptomatic groups. There were no plaques (0%) in the C5 ICA segment in the symptomatic group, whereas six plaques (6.7%) were located in this segment in the asymptomatic group. No statistically significant difference emerged.

**Table 1:** Comparison of baseline clinical characteristics between symptomatic and asymptomatic patients.

**Variables**	**Symptomatic (*n* = 19)**	**Asymptomatic (*n* = 89)**	***p*-value**
Sex: male (%)	12 (63.2)	61 (77.5)	0.788
Age (years)	71.2 ± 13.5	72.8 ± 14.4	0.768
Degree of stenosis (%)	67.4 ± 16.6	64.7 ± 15.1	0.577
Stenotic lesion			0.631
MCA M1 (%)	13 (68.4)	56 (62.9)	
ICA C1 (%)	0 (0)	3 (3.4)	
C2 (%)	2 (10.5)	9 (10.1)	
C3 (%)	1 (5.3)	1 (1.1)	
C4 (%)	3 (15.8)	14 (15.7)	
C5 (%)	0 (0)	6 (6.7)	

**Figure 3:**
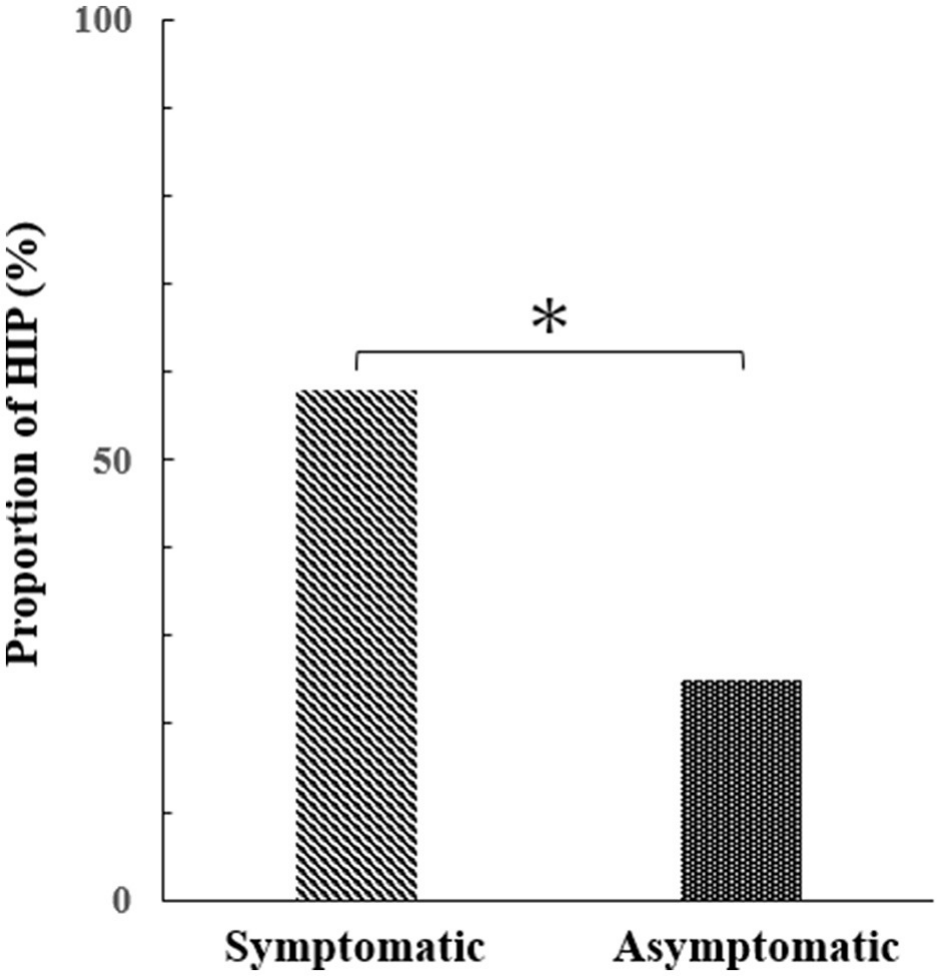
Difference in the percentage of HIPs between symptomatic and asymptomatic groups (Mann–Whitney test, *p* = 0.01*).

HIPs were detected in 11 of 19 plaques in the symptomatic group and 22 of 89 plaques in the asymptomatic group (57.9% and 24.7%, respectively). The proportion of HIPs in the symptomatic group was significantly higher than in the asymptomatic group (*P* = 0.01, Figure 3).

The mean SIplaque/SImuscle ratio was 1.52 ± 0.34 in symptomatic HIPs and 1.34 ± 0.27 in asymptomatic HIPs (*P* = 0.03). In the symptomatic group, the proportion of HIPs in each disease type is shown in Table 2. Notably, the proportion of HIPs in the A-to-A embolism group (85.7%) was higher than in the other groups (hemodynamic infarction: 0%, perforator infarction: 50%, undetermined lesions: 50%, TIA: 40%).

**Table 2:** Presence of HIPs in stroke subtypes.

**Stroke subtypes**	**Number of lesions**	**Number of HIP (%)**
A-to-A embolism	7	6 (85.7)
Hemodynamic infarction	1	0 (0)
Perforator infarction	4	2 (50)
Undetermined lesions	2	1 (50)
TIA	5	2 (40)

## Discussion

### Summary of key findings

This study demonstrated that HIPs were significantly more prevalent in symptomatic than in asymptomatic lesions among Japanese patients with ICAD. Within the symptomatic group, HIPs were most frequently observed in A-to-A embolism cases, compared to other stroke subtypes. These findings highlight the potential of DANTE T1-SPACE imaging to evaluate plaque instability and stratify the risk of ischemic stroke. Notably, HIP detection using this technique may also facilitate risk assessment in asymptomatic individuals. If confirmed by future prospective studies, HIPs identified by DANTE T1-SPACE could help guide decisions regarding intensified antithrombotic therapy or closer clinical follow-up. Thus, integrating DANTE T1-SPACE into routine clinical protocols may improve preventive strategies for stroke, including early intervention in high-risk but asymptomatic ICAD cases.

### Biological and clinical interpretation

ICAD is a major cause of ischemic stroke and is reported to account for up to 50% of cases in Asia (Wong, 2006). Accordingly, the evaluation of ICAD is critically important in the clinical diagnosis of cerebral infarction in Asian populations. Efforts to detect intracranial plaques using various imaging modalities, including contrast-enhanced MRI, have been ongoing for decades (Aoki et al., 1995). The advent of high-resolution MRI and the widespread adoption of 3T systems have enabled better visualization of ICAD plaques, leading to a growing number of studies in recent years (Mandell et al., 2017). In this context, we applied a novel black-blood technique using DANTE pulses, which enhance vessel wall delineation by attenuating residual blood and cerebrospinal fluid signals (Xie et al., 2016; Wang et al., 2016; Fushimi et al., 2022). Using this sequence, we investigated the association between ischemic events and plaque characteristics, focusing on HIPs. Hyperintensity of cervical carotid plaques on T1-weighted MRI has been reported to reflect IPH (Yoshida et al., 2008; Albuquerque et al., 2007; Bitar et al., 2008), which is associated with ischemic events (Takaya et al., 2006; Yamada et al., 2007; Altaf et al., 2007, 2008; Kurosaki et al., 2011). HIPs in ICAD may similarly reflect IPH and are increasingly recognized as a marker of plaque vulnerability in both anterior (Xu et al., 2012) and posterior circulations (Yu et al., 2015; Zhu et al., 2018). IPH can promote thrombus formation and embolic stroke through inflammation, neovascular leakage, or fibrous cap rupture—mechanisms well-established in extracranial carotid artery disease and now being recognized in ICAD. Our findings—showing a higher prevalence of HIPs in symptomatic plaques, particularly those linked to A-to-A embolism—support the clinical relevance of HIPs as non-invasive surrogates of biologically unstable plaques in the intracranial circulation.

### Comparison with regional and international literature

As shown in Table 3, the proportion of HIPs in symptomatic lesions in our study was 57.9%, notably higher than the 19.6% and 26.8% reported by Xu et al. (2012) and Yang et al. (2014), respectively. This discrepancy may stem from differences in MRI sequences, plaque definitions, and study populations. While those studies investigated Chinese patients, our cohort consisted of Japanese individuals. Ethnic and demographic factors may influence plaque composition and morphology (Kuller, 2004; Rockman et al., 2013). For example, ICAD is more common and often more symptomatic in East Asian populations compared to Western cohorts (Wang et al., 2019), potentially influencing baseline plaque characteristics and vulnerability. In addition, a meta-analysis by Sun et al. (2020) reported that the RNF213 rs112735431 variant—associated with ICAD and moyamoya disease—conferred a slightly lower risk in Chinese populations compared to Japanese and Korean populations, suggesting possible genetic differences in ICAD susceptibility among East Asians. In addition, studies from Western countries have shown the utility of vessel wall MRI in diverse populations. Mossa-Basha et al. (2016) and Mandell et al. (2017) emphasized the value of high-resolution vessel wall imaging for identifying high-risk plaques in Western cohorts. These findings imply that while population differences exist, imaging biomarkers such as HIPs may hold universal relevance for assessing intracranial plaque vulnerability across geographic and ethnic settings.

**Table 3:** Comparison among previous studies about HIPs of ICAD in the anterior circulation.

**Previous studies**	**Modality/sequence**	**Definition of HIP**	**Evaluation method**	**Percentage of HIP**
Xu et al. (2012)	3T MRI/T1-weighted fat-suppressed	(Plaque SI)/(adjacent muscle SI) > 1.5	>70% MCA stenosis (area stenosis), symptomatic vs. asymptomatic	Symptomatic: 19.6% Asymptomatic: 3.2%
Yang et al. (2014)	3T MRI/double inversion recovery (T1)	(Plaque SI)/(SI of adjacent gray matter) > 1.5	>50% MCA stenosis (area stenosis), symptomatic vs. asymptomatic	Symptomatic: 26.8% Asymptomatic: 0%
Wu et al. (2018)	3T MRI/ inversion-recovery prepared SPACE	(Plaque SI)/(SI of reference vessel wall) > 1.5	Any degree of symptomatic ICA and MCA stenosis (area stenosis), A-to-A embolism vs. non-A-to-A embolism	A-to-A embolism: 75% Non-A-to-A embolism: 21%
This study	3T MRI/ DANTE T1-SPACE	(Plaque SI)/(SI of ipsilateral temporal muscle) > 1.5	>40% ICA and MCA M1 stenosis (diameter stenosis), symptomatic vs. asymptomatic	Symptomatic: 57.9% Asymptomatic: 24.7% A-to-A embolism: 85.7%

### Technical innovations related to DANTE preparation

A new BB technique using DANTE pulses allows for more detailed evaluation of intracranial vessel walls (Xie et al., 2016; Wang et al., 2016; Fushimi et al., 2022). This approach significantly improves flow suppression, especially in complex regions like the carotid siphon, where residual flow-related signals from conventional SPACE sequences can mimic plaques. DANTE pulses attenuate slow and turbulent flow signals, enhancing the black-blood effect and vessel wall contrast. This technical innovation addresses one of the main limitations of conventional vessel wall MRI and has been shown to be especially useful in pre-contrast protocols (Xie et al., 2016). In our study, the integration of DANTE into the T1-SPACE sequence likely improved plaque visualization and contributed to the higher detection rate of HIPs. These refinements may enhance diagnostic reliability and facilitate more accurate identification of vulnerable plaques in clinical practice. Additionally, compared to previous studies that employed multiparametric vessel wall imaging with pre- and post-contrast sequences, our use of a pre-contrast DANTE T1-SPACE protocol may limit direct comparability. While this method is advantageous for suppressing blood signal artifacts and detecting HIPs, it may be less sensitive to features such as contrast enhancement or fibrous cap integrity. These methodological differences should be considered when comparing results across studies and highlight the importance of standardization in vessel wall imaging protocols.

### Limitations and future directions

This study has several limitations. First, the retrospective design and strict inclusion criteria resulted in a relatively small sample size, with potential selection bias favoring patients with better image quality or available follow-up data. Second, pathological validation of HIPs remains limited in ICAD. While histopathological correlation between T1 hyperintensity and IPH is well-established in extracranial carotid plaques, such validation is rarely feasible for intracranial arteries due to the difficulty in obtaining tissue samples. Nonetheless, prior autopsy studies, such as that by Chen et al. (2008), have demonstrated that intracranial plaques in infarcted brains are more likely to contain IPH and thrombus, supporting the biological plausibility of our findings. Another limitation is that our analysis was restricted to pre-contrast T1-weighted images, without incorporating plaque morphology, contrast enhancement, or T2-weighted signal. These additional parameters may further refine the assessment of plaque vulnerability and should be considered in future studies using multiparametric vessel wall imaging. Furthermore, our study focused only on the anterior circulation, where image reproducibility is higher; future investigations should extend these evaluations to the posterior circulation to better capture the full spectrum of ICAD pathology. Moreover, this was an exploratory study, and no adjustment was made for multiple comparisons. The results, particularly those of subgroup analyses, should therefore be interpreted with caution and warrant confirmation in larger prospective cohorts. Finally, the definition of HIPs was based on the cervical carotid plaque criteria, as in previous reports (Xu et al., 2012; Yang et al., 2014; Wu et al., 2018), due to the lack of histological correlation in ICAD. Although this approach provides consistency with prior literature, it remains unclear whether the same definition accurately reflects IPH in intracranial lesions. Future studies with histopathological validation and multiparametric imaging approaches are needed to establish the clinical and biological significance of HIPs in ICAD.

## Conclusion

Taken together, our findings support HIPs—as revealed by high-resolution DANTE T1-SPACE—as promising imaging biomarkers of symptomatic plaque and stroke risk in ICAD. By integrating advanced flow-suppressed imaging with detailed stroke-subtype analysis and situating our results within a broad international context, this study offers new insight into non-invasive risk stratification for patients with intracranial stenosis.

## Data Availability

The original contributions presented in the study are included in the article/supplementary material, further inquiries can be directed to the corresponding author.
